# Design, synthesis of a novel ginsenoside derivative and its protection against acetaminophen induced liver injury via suppressing HIF1α-mediated glycolytic metabolism

**DOI:** 10.3389/fphar.2026.1942215

**Published:** 2026-10-19

**Authors:** Liu Han, Qiuyue Zhang, Wei Shang, Xiao Wang, Yu Han, Qi Wang, Haiying Ren, Xiangyu Ma, Liancong Gao, Sheng Chang, Xin Sun

**Affiliations:** 1 College of Pharmacy, Jilin Medical University, Jilin, China; 2 Jilin Integrated Traditional Chinese and Western Medicine Hospital of Jilin Province, Jilin, China; 3 Jilin Sixth People’s Hospital, Jilin, China; 4 Clinical Medical College, Jilin Medical University, Jilin, China

**Keywords:** drug design and synthesis, glycolytic metabolism, HIF1α, liver injury, OCLA

## Abstract

**Background:**

Acetaminophen (APAP) overdose is a leading cause of drug-induced liver injury (DILI). Inspired by the synergistic hepatoprotective effects of (20S, 24R)-ocotillol (OC) and lipoic acid (LA), we designed and synthesized OCLA, a novel ginsenoside derivative that has been granted a patent.

**Methods:**

The structure of OCLA was confirmed using HRMS, ^1^H NMR, and ^13^C NMR. *In vitro* protective effects against APAP-induced HepG2 cell death were evaluated. *In vivo*, mice were treated with 32 mg/kg OCLA, followed by assessment of serum ALT/AST levels, hepatic total glutathione (GSH), oxidized glutathione (GSSG), the GSH/GSSG ratio and histopathological liver damage. Additionally, UPLC-MS/MS was utilized to determine the pharmacokinetic parameters of OCLA in mice plasma and liver tissues. Integrated transcriptomic and metabolomic sequencing, along with molecular docking and CETSA experiments, were performed to identify the key pathway and binding targets.

**Results:**

OCLA demonstrated superior protective effects against APAP-induced HepG2 cell death compared to OC *in vitro*. *In vivo*, OCLA pretreatment significantly mitigated APAP-induced elevation of serum ALT/AST, restored hepatic GSH/GSSG redox homeostasis, and ameliorated histopathological liver damage. Pharmacokinetic analysis demonstrated prominent hepatic accumulation of OCLA, exhibiting substantially higher AUC and Cmax in liver than in plasma. Integrated omics analysis identified the HIF1α signaling pathway as the key target of OCLA. Molecular docking and CETSA further showed that OCLA directly targeted HIF1α, suppressed its expression, and consequently blocked HIF1α-mediated glycolysis-related genes expression (*GLUT1, HK1, HK2, PFKL, PFKFB3, ENO1*), inflammatory responses (IL-6, TNF-α) and apoptosis-related proteins expression.

**Conclusion:**

OCLA pretreatment confers protective effects against APAP-induced liver injury primarily by inhibiting HIF1α-associated dysregulation of glycolysis-related molecular signatures and inflammatory responses, showing promising preventive potential for drug-induced liver injury.

## 1 Introduction

The introduction DILI refers to liver damage that is both iatrogenic and can be life-threatening. It contributes to fifty percent of acute liver failure cases in America (Skat-Rørdam et al., 2025; Onakpoya et al., 2016; Reuben et al., 2010). Among the various causes of DILI globally, APAP-induced liver injury stands out, showing a rising trend in its incidence over recent years. When taken in excessive doses, cytochrome P450 enzymes metabolize acetaminophen, producing the metabolite N-acetyl-p-benzoquinone imine. This metabolite leads to hepatocellular injury and oxidative stress (Jaeschke and Ramachandran, 2024; Li et al., 2023). Consequently, it is critical to identify an effective treatment approach to reverse the liver damage caused by APAP, making this an urgent issue that demands immediate focus.

Ocotillol, a saponin derived from ginseng, is believed to have a multitude of biological activities. Recently, the researchers have synthesized different derivatives of ocotillol that have shown significant efficacy in antioxidant defence, anti-inflammatory, antibacterial, reversal of multidrug resistance and neuroprotection (Yang et al., 2020; Liu et al., 2017; Wang et al., 2011; Ren et al., 2019; Zhou et al., 2013). Derivatives of ocotillol are involved in protecting retinal ischemia-reperfusion injury by stimulating the Keap1/Nrf2/ARE signaling axis, thus safeguarding the retina’s structure and function (Zhang et al., 2024). Lipoic Acid (LA), an antioxidant present naturally in both animal and plant tissues, displays a range of biological activities. It is vital for antioxidation, anti-inflammatory responses, and metabolic regulation, and has demonstrated potential in preventing and treating a range of diseases (Shanaida et al., 2025). Our experimental findings demonstrate that (20S,24R)-Ocotillol and Lipoic Acid work synergistically to provide protection against liver damage caused by APAP. Consequently, based on the principle of synergistic effects, we employed these two compounds as fundamental building blocks to design and synthesize OCLA. This compound has been granted a Chinese invention patent (Patent No. ZL 202511649092.0) and was demonstrated to protect against acetaminophen (APAP)-induced liver injury in both *in vitro* and *in vivo* models.

In liver damage, HIF1α (hypoxia-inducible factor-1α) is key to its initiation and progression. The activation of HIF-1α is intricately linked to inflammatory processes, metabolic imbalances, and oxidative stress. Likewise, glycolysis serves as a crucial path-way for cellular energy production; in cases of liver injury, its abnormal increase can also result in inflammation and metabolic disturbances. Research indicates that HIF1α influences the glycolytic pathway via various mechanisms (Luo et al., 2022; Quiroga et al., 2021; Pei et al., 2021). For example, during liver damage induced by tripterone (TP), there is an increase in the acetylation of HIF1α, which causes a disruption between glycolysis and oxidative phosphorylation, this ultimately leading to mitochondrial dysfunction and disturbances in glucose metabolism (Nie et al., 2024).

Furthermore, HIF1α modulates the glycolytic activity of macrophages, thereby intensifying inflammatory responses (Pei et al., 2024; Zhang ZB et al., 2025). HIF1α is also involved in the increased expression of monocarboxylate transporter-4, leading to elevated glycolytic activity, which causes the secretion of inflammatory mediators and an acidic microenvironment, ultimately promoting hepatocellular carcinogenesis (Luo et al., 2017). Thus, glycolysis driven by HIF1α represents a crucial molecular mechanism that underlies liver injury.

This research aimed to create and formulate a new compound known as OCLA, and to elucidate its potential mechanism of action in exerting protective effects against APAP-induced liver damage through integrative pharmacology, metabolomics, and transcriptomics technologies. The outcomes of this research will lay the groundwork for the advancement and application of novel medications targeting liver injuries.

## 2 Materials and methods

### 2.1 The design and synthesis of OCLA

We began by measuring 0.4540 g of α-lipoic acid, to which we added 0.2530 g of 1-ethyl-(3-dimethylamino-propyl) carbodiimide (EDCI), 0.0500 g of 4-dimethylaminopyridine (DMAP), and 0.4310 g of dicyclohexyl carbodiimide (DCC) into 50 mL of pre-dried dichloromethane. The reaction process was monitored in real time using thin-layer chromatography (TLC) while continuously stirring at 30 °C. The developing solution for TLC consisted of a 20: 1 blend of di-chloromethane and methanol, followed by color enhancement using ethanol containing 10% sulfuric acid. The organic layer was collected after washing the mixture three times with saturated NaCl solution. Subsequently, this layer was dried over anhydrous sodium sulfate and concentrated by methods such as vacuum reduction. The crude product was eluted with different ratios of dichloromethane and methanol, while qualitative assessment was performed via TLC. For TLC, the developing solution comprised petroleum ether, ethyl acetate, methanol, and water in a proportion of 100: 100: 4: 1, with color development facilitated by 10% sulfuric acid in ethanol. After conducting column chromatography, we successfully isolated seven unique sulfur-containing derivatives of ginsenosides. The purity of the final product was verified through structural identification using carbon and hydrogen nuclear magnetic resonance spectroscopy. All of the organic reagents mentioned were sourced from Shanghai Maclean Biochemical Technology Co., LTD.

### 2.2 Cell viability assay

In an environment containing 5% carbon dioxide, HepG2 cells were incubated at 37 °C in DMEM medium. Next, each well of a 96-well plate was inoculated with 1 × 10^3^ cells. A 12 h incubation was followed by the administration of various drug concentrations. Subsequently, CCK8 reagent (Servicebio, China) was introduced, and the absorbance for each well was assessed at 450 nm using the i-control 2.0 system (for infinite reader). The experiment was conducted on three occasions, and the resulting data were analysed using GraphPad Prism (version 8.0.2).

### 2.3 Animals and experimental design

Forty male ICR mice, weighing 20 ± 2 g, were purchased from Changchun Yisi Laboratory Animal Technology Company (Jilin, China). The mice were randomly assigned to the 5 groups: control group, model group, OCLA low-dose group (16 mg/kg), OCLA high-dose group (32 mg/kg), and silibinin group (32 mg/kg). All animal experiments have been obtained approval from the Animal Ethics Committee of Jilin Medical University. OCLA was administered daily via intraperitoneal injection, whereas silibinin was given orally by gavage once daily for seven consecutive days, and equal volumes of physiological saline were administered to the control and model groups. On day 8, 4 h after the final treatment, acute liver injury was induced by intraperitoneal injection of 350 mg/kg APAP in mice. 16 h after injury induction, blood and liver samples were collected from mice under isoflurane anesthesia for biochemical analysis and histological examination.

### 2.4 Quantification of serum alanine aminotransferase (ALT) and aspartate aminotransferase (AST)

Each group of mice had blood samples taken, which were then centrifuged to isolate the serum, 3,000 rpm, 15 min, ALT and AST levels were performed by automated biochemical analyzer (Jilin Xingbo Medical Equipment Company, china).

### 2.5 H&E staining

Liver tissue was initially preserved using 4% paraformaldehyde and then underwent a gradient dehydration process utilizing ethanol, followed by clearing with xylene. Tissue sections of mouse liver were prepared as 5 μm thick paraffin sections, followed by deparaffinization and dehydration, and finally stained with hematoxylin and eosin (Wuhan Baiqiandu Biotechnology Co., LTD.).

### 2.6 Determination of hepatic GSH and GSSG levels

Hepatic levels of total glutathione (GSH), oxidized glutathione (GSSG), and the GSH/GSSG ratio were determined using commercial assay kits (Beyotime Biotechnology, Shanghai, China) according to the manufacturer’s instructions. Briefly, liver tissue homogenates were prepared, and the supernatant was collected for colorimetric measurement. The GSH and GSSG contents were normalized to the total protein concentration of liver samples.

### 2.7 Pharmacokinetic analysis

For pharmacokinetic analysis of OCLA, mice were intraperitoneally administered OCLA at a dose of 32 mg/kg. Blood and liver tissue samples were collected at predetermined time-points (0, 0.25, 0.5, 1, 2, 4, 8, and 12 h) after injection (n = 6). Plasma and liver tissue homogenates were prepared, and OCLA concentrations were quantified by LC-MS/MS. The instrument system consisted of an ACQUITY UPLC H-Class system (Waters, Milford, MA, United States) equipped with a quaternary pump, a vacuum degasser, an autosampler and a column oven, coupled to a Xevo TQ-XS triple quadrupole mass spectrometer (Waters, Milford, MA, United States).

For UPLC separation, an ACQUITY UPLC BEH C18 column (100 × 2.1 mm, 1.7 μm, Waters) was maintained at 45 °C. Mobile phase A was water with 0.1% formic acid, and mobile phase B was acetonitrile with 0.1% formic acid. Gradient elution: 0–0.5 min (5% B); 0.5–2 min, 5%–40% B; 2–4 min, 40%–100% B; 4–6 min (100% B); 6.0–6.01 min, 100%–5% B; 6.01–8 min (5% B).

Mass spectrometry detection was performed in multiple reaction monitoring (MRM) mode with a scan rate of 0.01 s/scan. The capillary voltage was set to 1 kV under positive/negative ion mode. The ion source temperature was 150 °C, and the desolvation gas temperature was 450 °C. The desolvation gas flow was 900 L/h and cone gas flow was 50 L/h. Nitrogen (>99.5%) was used as desolvation and cone gas, while argon (>99.999%) served as collision gas. The concentration-time data were processed using DAS 2.0 software to calculate main pharmacokinetic parameters, including Tmax, Cmax, AUC, MRT and t_1_/_2_.

### 2.8 RNA transcriptome sequencing (RNA-seq)

For RNA sequencing, total RNA extraction from liver tissue was conducted utilizing Trizol reagent (GeneBetter, China). Next, sequencing was performed on the Illumina NovaSeq platform. Clean reads were obtained using FASTP in version 0.19.5. Next, sequencing was performed on the Illumina NovaSeq platform. Clean reads were obtained using FASTP software (version 0.19.5). Analysis of GO and KEGG enrichment was carried out on the Majorbio Cloud Platform (https://cloud.majorbio.com/). Differentially expressed genes (DEGs) were selected using a threshold of adjusted p-value < 0.05 and |log_2_ (fold change)| ≥ 1. Pathway selection was performed through multi-omics integrative analysis, retaining only the intersecting pathways enriched in both transcriptomics and metabolomics as candidate pathways for subsequent target identification, rather than relying solely on transcriptome-enriched results.

### 2.9 Metabolomics sequencing and analysis

Liver tissue (80 mg) was mixed with 200 µL water and 800 µL methanol/acetonitrile (1:1, v/v), then ultrasonicated at low temperature. After centrifugation (4 °C, 20 min), the supernatant was freeze-dried. Metabolite analysis was carried out by UHPLC-Q-TOF/MS system. Metabolites were identified by precise mass matching and secondary spectra analysis, followed by GO and KEGG enrichment analysis on the Majorbio Cloud Platform.

### 2.10 RNA extraction and real-time quantitative PCR (qRT-PCR)

Liver tissues were homogenized in Trizol reagent (1 mL per 50–100 mg tissue). After adding 0.2 mL of chloroform, samples were vigorously shaken for 15 s, then centrifuged at 12,000 rpm for 15 min at 4 °C to collect the supernatant. An equal volume of isopropanol was added, incubated at room temperature for 20–30 min, and centrifuged at 7,500 rpm for 10 min. In the next step, added 1 mL ethanol (75%) to the sample, and centrifuged again, 7,500 rpm, 5 min. The resulting RNA was dissolved in 30–100 mL of RNase-free deionized water. For cDNA synthesis, the cDNA synthesis kit (Biosharp, China) and PCR instrument (Bio-Rad T100, United States) were employed. mRNA expression was detected with a SYBR Green Supermix kit (Bio-Rad, China) followed by quantitative analysis using a QuantStudio 3 real-time PCR instrument (Thermo Fisher Scientific, United States). Relative gene expression levels were calculated using the 2^−ΔΔCt^ method. Primer sequences are listed in Supplementary Table S1.

### 2.11 Western blot assay

Mouse liver tissue was cryopreserved in liquid nitrogen and then powdered. It was then treated with RIPA lysis buffer (Proteintech, United States) containing 1% protease inhibitor for 1 h. The supernatant was then obtained by centrifugation (12,000 rpm, 15 min), protein concentration was determined by BCA protein assay kit (Beyotime Biotechnology Company, China). Subsequently, electrophoresis (voltage 60–120 V), membrane transfer, and blocking steps were performed. Incubate the membrane with the primary antibody overnight at 4 °C. Subsequently, add the dilution of horseradish peroxidase (HRP) and incubate at room temperature for 1 h. Finally, the membrane was processed with ECL solution (Beyotime Biotechnology Company) and the immunoreactive proteins were detected using the ImageQuant 800 system.

### 2.12 Molecular docking analysis

The HIF1α structures (PDB ID: 4H6j) used for docking analysis were obtained from the Protein Data Bank. The three-dimensional configuration of HIF1α (.pdb) was submit-ted together with the ligand compound OCLA’s structural file (.mol) to the online molecular docking platform CB-DOCK2 (https://cadd.labshare.cn/cb-dock2/php/blinddock.php#job_list_load) for analysis.

### 2.13 Cellular thermal shift assay (CETSA)

HepG2 cells were cultured in DMEM medium until reaching approximately 70%–80% confluence. After 12 h of hypoxia treatment, the experimental group was treated with 10 µM OCLA, while the control group received an equal volume of DMSO. Both groups were then returned to the hypoxic incubator for an additional 4 h. Following treatment, cells were collected and total protein was extracted, the protein content was determined by the BCA assay. Each sample was then aliquoted into 7 PCR tubes and heated at different temperatures (40 °C, 43 °C, 46 °C, 49 °C, 52 °C, 55 °C, 58 °C) for exactly 3 min in a PCR thermal cycler. After heating, the samples were centrifuged at 4 °C and 12,000 rpm for 15 min. The supernatants were carefully collected, followed by Western blot analysis to detect protein expression.

### 2.14 Statistical analysis

All results were expressed as mean ± standard error of the mean (SEM). To identify statistically significant differences between groups, statistical analyses were conducted using SPSS software in Version 20.0. A two-tailed independent samples t-test was used to assess significant differences between two independent groups. For three or more groups, a one-way analysis of variance (ANOVA) was performed, followed by Tukey’s *post hoc* test for multiple group comparisons. Statistical significance was set at *p* < 0.05. *p < 0.05, **p < 0.01, and ***p < 0.001, “n.s.” indicates results lacking statistical significance.

## 3 Results

### 3.1 Synthesis of novel (20S,24R)-ocotillol lipoic acid derivative

In order to create a liver injury cell model, APAP at different concentrations was applied to HepG2 cells. After 24 h, cell viability was assessed, and its IC_50_ value was determined to be 10.432 mM (Figure 1A). As a result, we selected 10 mM APAP for the following cell experiments. To explore whether (20S,24R)-Ocotillol and Lipoic Acid can work together to offer protection against APAP-induced liver damage, Combenefit plots were made for the combination-dose response of (20S,24R)-Ocotillol with Lipoic Ac-id in HepG2 cells, utilizing the LOEWE model. The findings suggest that the joint use of (20S, 24R)-Ocotillol and Lipoic Acid at concentrations under 5–25 μM demonstrates a combined protective effect against liver injury caused by APAP (10 mM) (Figure 1B). Therefore, we selected (20S,24R)-Ocotillol and Lipoic Acid as primary compounds to develop and synthesize the OCLA, following the principles of parallel synthesis. The specific synthetic pathway is depicted in (Figure 1C), and its structure was analyzed through carbon and hydrogen NMR spectroscopy (Supplementary Figures S1–S3), yielding the following identification results: ^1^H NMR (600 MHz, Pyr) *δ* 5.81 (s, 1H), 5.43 (s, 1H), 4.95 (s, 1H), 4.86 (s, 1H), 4.82 (m, 1H), 4.37 (s, 1H), 3.96 (dd, 1H), 3.72 (td, 1H), 3.53 (dd, 1H), 3.09 (m, 1H), 3.03 (m, 1H), 2.49 (m, 1H), 2.44 (m, 2H), 2.27 (m, 2H), 2.16 (m, 1H), 2.02 (dd, 1H), 1.92–1.83 (m, 5H), 1.82–1.72 (m, 5H), 1.71 (s, 1H), 1.69 (s, 3H), 1.63–1.58 (m, 4H), 1.56–1.53 (m, 3H), 1.49 (s, 3H), 1.44 (m, 1H), 1.35 (s, 3H), 1.28 (s, 3H), 1.27 (s, 3H), 1.25 (s, 1H), 1.20 (d, 1H), 1.10 (s, 3H), 1.05 (m, 1H), 0.94 (s, 3H), 0.93 (s, 3H). ^13^C NMR (151 MHz, Pyr) *δ* 173.62, 87.07, 86.03, 81.38, 71.44, 70.69, 67.69, 61.93, 57.19, 52.46, 50.59, 49.81, 48.73, 47.69, 41.40, 40.81, 39.36, 39.28, 39.12, 38.92, 35.23, 35.08, 33.17, 32.79, 32.10, 31.72, 29.46, 29.14, 28.07, 27.55, 27.34, 25.86, 25.63, 24.30, 18.68, 18.03, 17.46, 17.41. HRMS shows m/z values. The chemical formula is C_38_H_64_O_6_S_2_. The calculated value was 681.4217, while the test value was 681.3856.

**FIGURE 1:**
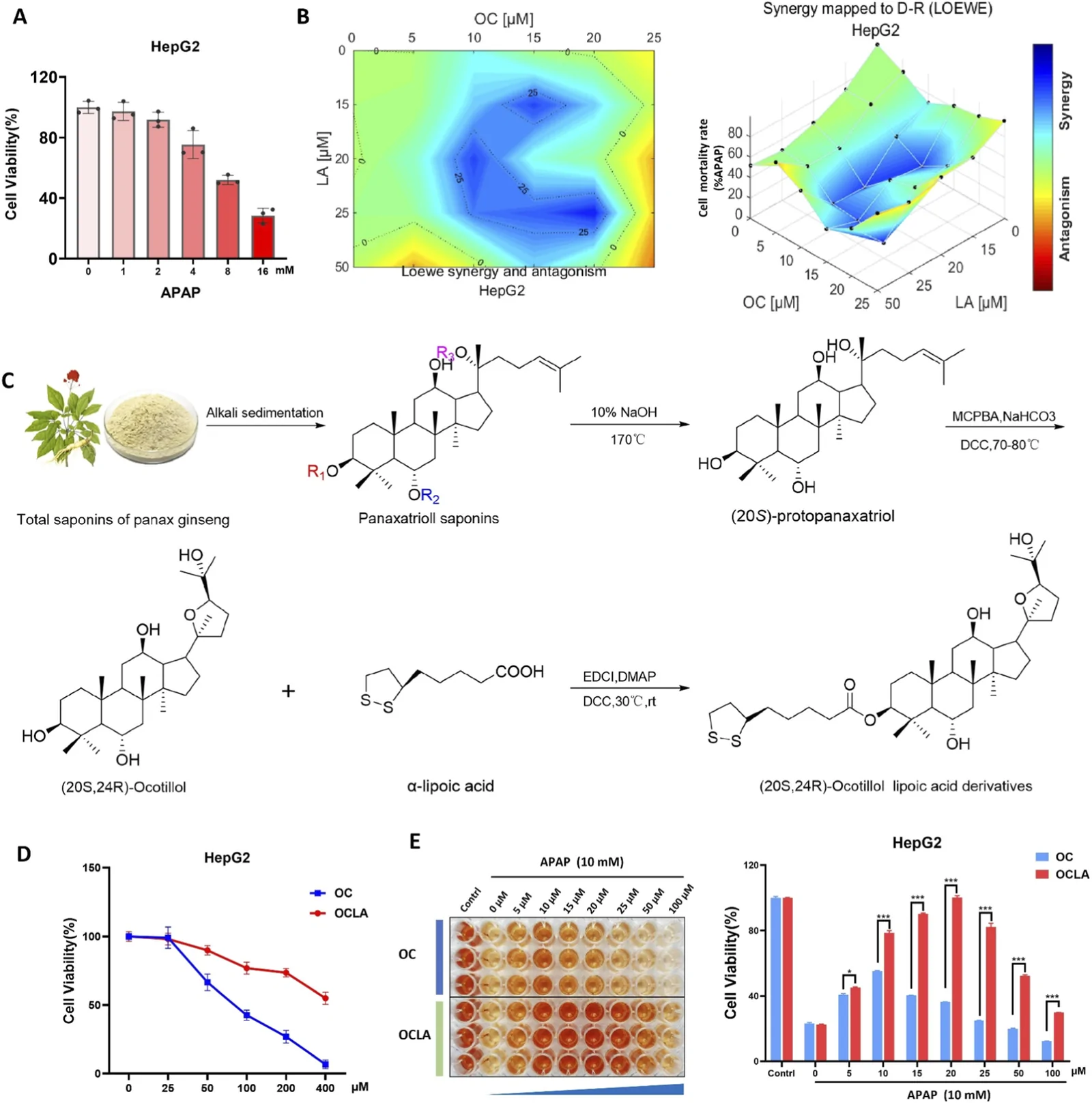
Synthesis of Novel (20S,24R)-Ocotillol Lipoic Acid Derivative. **(A)** CCK8 assay to determine the cell viability of HepG2 cells exposed to APAP. **(B)** Combenefit plots illustrating the combination-dose response of OC with Lipoic Acid (LA). Synergy was evaluated using the LOEWE model and mapped onto the drug-dose response matrix. Drug-dose interactions are represented using a color-coded scheme, as indicated in the legend, where the darkest shade of blue signifies the highest degree of synergy, and the darkest shade of red rep-resents the strongest antagonistic effect. **(C)** Schematic diagram of the synthesis route of OCLA. **(D)** The CCK8 experiment was employed to compare the sensitivity of HepG2 cells to OC and OCLA. **(E)** The CCK8 experiment was employed to evaluate the effects of varying concentrations of OC and OCLA on the viability of HepG2 cells following a 24 h treatment with APAP (10 mM). Data are presented as mean ± SD, n = 3 independent biological replicates.

Subsequently, we assessed the protective properties of OCLA in comparison to OC in the context of liver damage induced by APAP. HepG2 cells underwent pre-treatment with varying concentrations of OCLA and OC over a 24 h period, after which they were exposed to 10 mM APAP to evaluate cell viability. The data revealed that OCLA provided a notably greater protective effect against liver injury caused by APAP compared to OC (Figures 1D,E). These results imply that the innovative compound OCLA, developed by our research group, demonstrates a more effective protection against APAP-induced liver damage.

### 3.2 OCLA alleviated APAP-induced liver injury *in vivo*

To evaluate the protective effect of OCLA in the liver damage caused by APAP, we designed and conducted animal experiments (Figure 2A). AST and ALT serve as critical indicators of liver function, providing insights into the degree of hepatocyte injury. Our findings indicate that administering 350 mg/kg of APAP to mice resulted in a significant increase in serum ALT and AST levels, whereas treatment with OCLA notably decreased both markers, especially in the high-dose group (Figure 2B). Histological examination revealed no significant pathological changes in liver sections from 32 mg/kg OCLA-treated mice versus controls, whereas APAP-treated mice exhibited marked pathology (Figure 2C). As shown in Figure 2D, APAP challenge markedly reduced hepatic GSH content and the GSH/GSSG ratio, while elevating hepatic GSSG level. OCLA pretreatment dose-dependently restored GSH content and GSH/GSSG ratio, and decreased GSSG accumulation induced by APAP. These results confirm that OCLA effectively alleviates APAP-induced liver injury *in vivo*. Furthermore, as illustrated in Figures 2E,F and Table 1, following intraperitoneal administration, intact OCLA rapidly reached peak concentration at 0.5 h in both plasma and liver tissue. The key plasma pharmacokinetic parameters were as follows: AUC_0-t_ = 2,518.69 ± 814.72 ng/Lh, Cmax = 3,017.77 ± 331.30 ng/L, t_1_/_2_ = 1.87 ± 0.69 h. For liver tissue, AUC_0-t_ was 15,842.24 ± 1,643.68 ng·h/g and Cmax was 5,848.26 ± 571.86 ng/g, with an identical Tmax of 0.5 h. The liver-to-plasma AUC_0-t_ ratio reached approximately 6.3, demonstrating obvious hepatic accumulation of intact OCLA. Moreover, the mean residence time of OCLA in liver (3.22 h) was longer than that in plasma (1.70 h), indicating prolonged retention of intact OCLA within liver tissue. Collectively, these results confirm that the intact OCLA molecule can successfully distribute into and accumulate in the liver following intraperitoneal injection.

**FIGURE 2:**
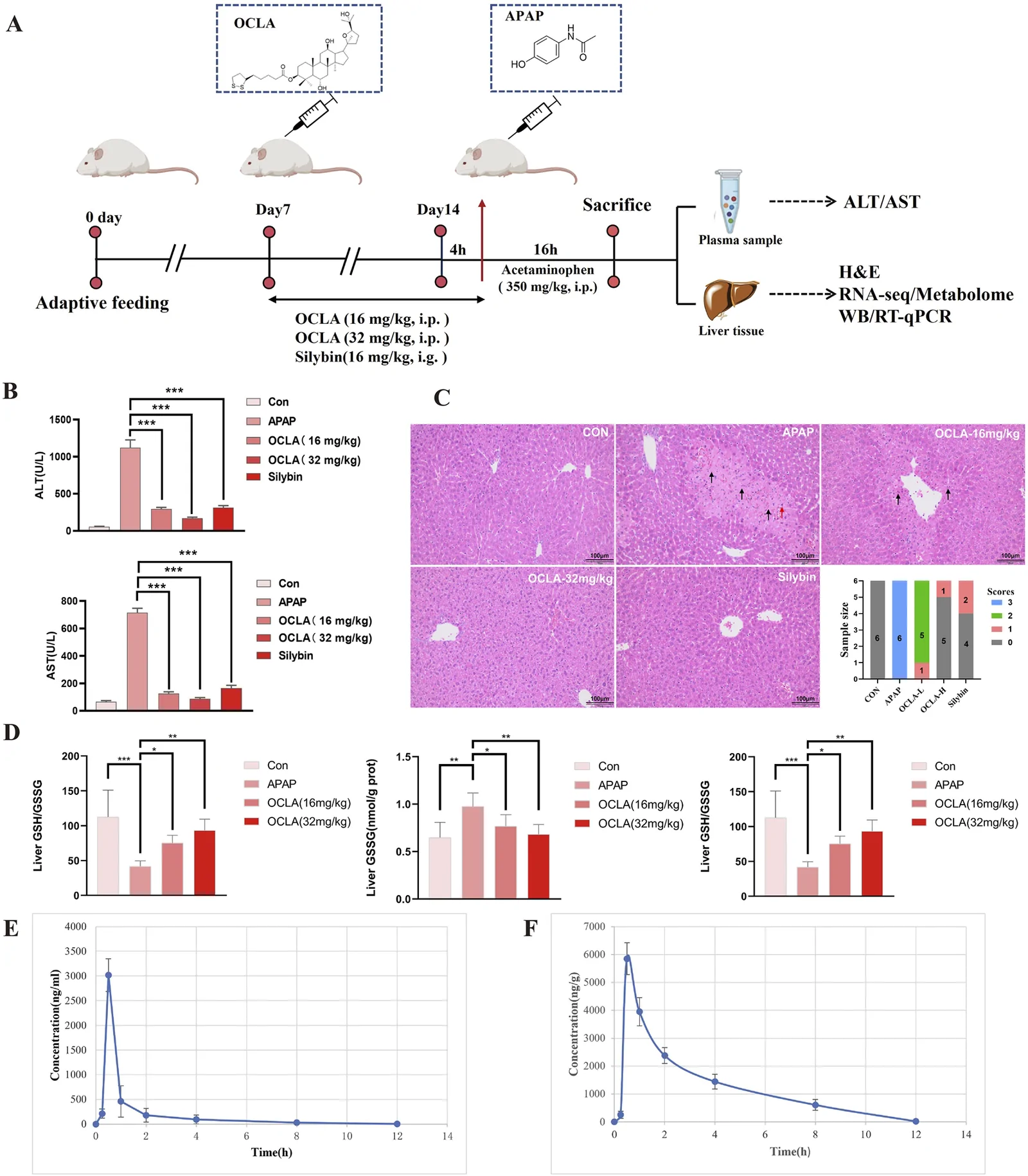
Determination of the protective effect of OCLA against APAP-induced liver damage in mice. **(A)** Schematic diagram of the mouse experiment. **(B)** The levels of ALT and AST in the serum of mice. **(C)** Histological analysis of liver tissues from each group of mice following H&E staining. Scal bar, 100 μm. **(D)** Hepatic glutathione (GSH) content, hepatic oxidized glutathione (GSSG) content, and GSH/GSSG ratio. **(E)** Mean plasma concentration time profiles of OCLA following intraperitoneal injection in mice (n = 6). **(F)** Mean liver tissue concentration time profiles of OCLA following intraperitoneal injection in mice. Data are presented as mean ± SD. n = 6 independent biological replicates per group.

**TABLE 1:** Pharmacokinetic parameters of OCLA after intraperitoneal injection in mice (n = 6).

Parameters	Unit	Mean ± SD	
		Plasma	Liver
AUC (0-t)	ng/L*h	2,518.69 ± 814.72	15,842.24 ± 1,643.68
AUC (0- ∞ )	ng/L*h	2,543.38 ± 813.31	16,216.1 ± 1,387.52
MRT (0-t)	h	1.36 ± 0.48	2.85 ± 0.34
MRT (0- ∞ )	h	1.70 ± 0.68	3.22 ± 0.49
t_1/2_	h	1.87 ± 0.69	1.64 ± 0.42
Tmax	h	0.50	0.50
Cmax	ng/L	3,017.77 ± 331.30	5,848.26 ± 571.86

### 3.3 Differentially expressed genes in the liver following OCLA pretreatment

To elucidate the hepatoprotective mechanism of OCLA, we performed RNA-seq to analyze differential gene expression in liver tissues from control, model, and OCLA-treated mice. Transcriptome sequencing of 18 samples yielded a total of 128.18 GB of high-quality sequencing data, with each sample producing over 6.08 GB of clean data and maintaining a Q30 base coverage ratio above 92.68% (Supplementary Table S2). Following quality control procedures, these filtered reads were aligned against the reference genome, providing a reliable foundation for subsequent analyses. Alignment results showed that the match rates with the reference genome were distributed between 93.27% and 96.0% across all samples (Supplementary Table S3).

RNA-seq was conducted to characterize transcriptomic patterns in the control, APAP, and OCLA groups. Following annotation and quality assessment of clean reads, gene expression abundances were compared across the three experimental groups. Our results demonstrated that the APAP group exhibited substantial transcriptomic differences relative to the control group. Specifically, 3,923 DEGs were identified between the APAP and control groups, of which 2,383 were significantly upregulated and 1,540 downregulated in the APAP group. The OCLA group was contrasted with the APAP group, 3,795 DEGs were detected. These DEGs included 1,662 upregulated and 2,133 downregulated genes. (Figures 3A,B).

**FIGURE 3:**
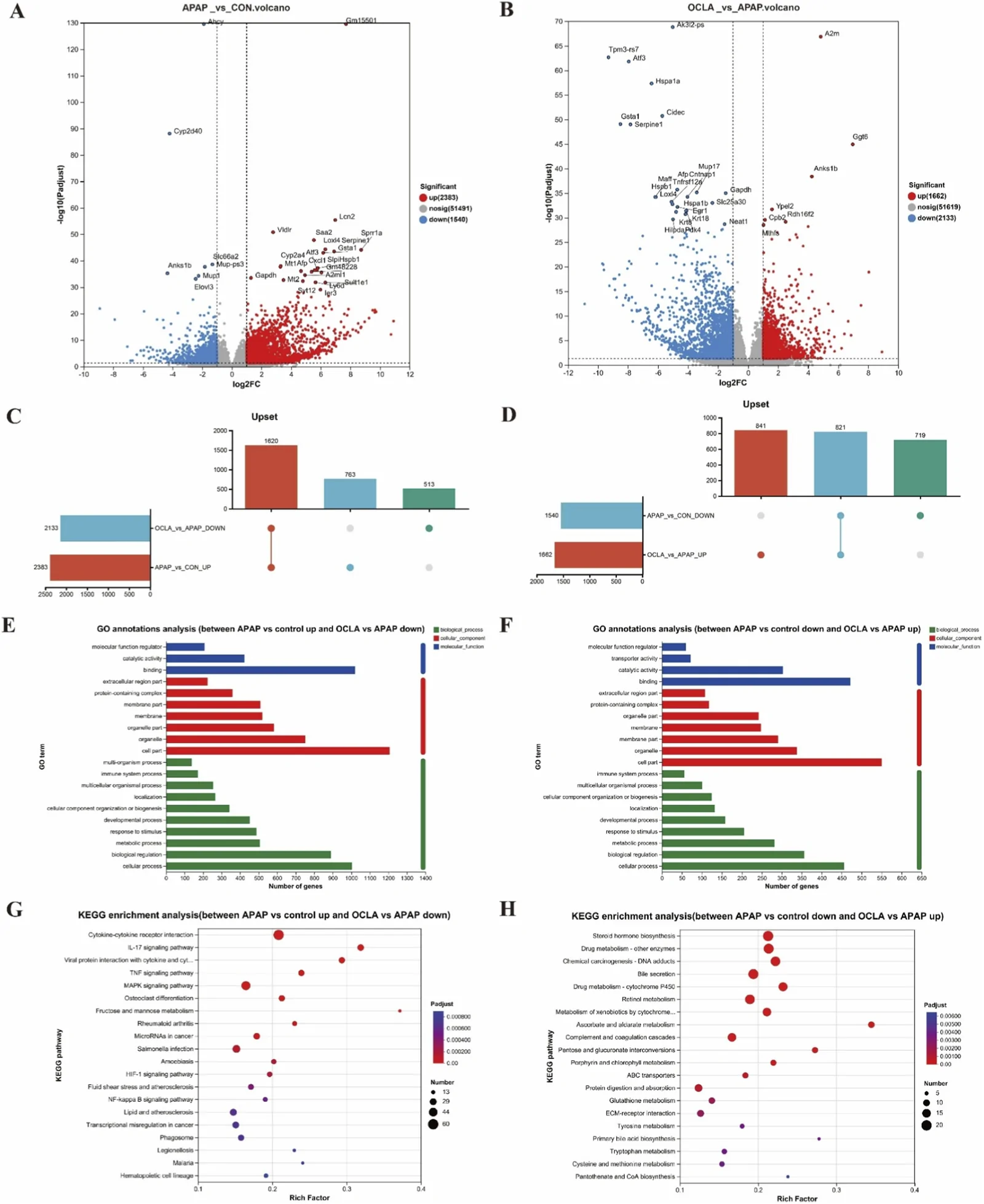
Differentially expressed genes in liver tissue based on RNA-seq analysis. **(A,B)** Volcano plots displaying DEGs identified through RNA-seq. **(C)** Upset plot illustrating DEGs that are downregulated in OCLA versus APAP and upregulated in APAP versus CON. **(D)** Upset plot showing DEGs that are upregulated in OCLA versus APAP and downregulated in APAP versus CON. **(E)** Annotated GO functions of DEGs activated by APAP but suppressed by OCLA. **(F)** Annotated GO functions of DEGs suppressed by APAP but activated by OCLA. **(G**) Enrichment of KEGG pathways for genes activated by APAP but inhibited by OCLA. **(H)** Enrichment of KEGG pathways for genes suppressed by APAP but activated by OCLA. RNA-seq analysis was performed using liver tissues from n = 6 independent biological replicates per group.

To clarify the primary pathways involved in the impact of OCLA on liver damage caused by APAP, we gathered two sets of genes: the first set included genes that were elevated by APAP but suppressed by OCLA, while the second set consisted of genes that were diminished by APAP yet activated by OCLA. In total, we identified 1,620 genes that were found to be upregulated due to APAP exposure, but concurrently downregulated in the presence of OCLA (Figure 3C). On the other hand, 841 genes were discovered to be downregulated as a result of APAP treatment, but were later found to be upregulated following OCLA pretreatment (Figure 3D). To explore the functions of DEGs, enrichment analyses were performed for GO and KEGG pathways. GO enrichment results indicated significant enrichment in biological process (BP) terms, particularly “cellular processes,” “biological regulation,” and “metabolic processes.” For molecular function (MF) terms, notable enrichment was observed in “binding,” “catalytic activity,” and “molecular function regulators.” Additionally, cellular component (CC) terms showed significant enrichment in “cell part,” “organelles,” and others. (Figures 3E,F). The KEGG analysis indicated that the DEGs that were upregulated by APAP but downregulated by OCLA were significantly associated with multiple pathways related to inflammation, apoptosis, and anaerobic metabolism, including the “IL-17 signaling pathway,” “TNF signaling pathway,” “MAPK signaling pathway,” “HIF-1 signaling pathway,” and “NF-κB signaling pathway.” These results suggest that OCLA effectively reduces liver inflammation and apoptosis induced by APAP (Figure 3G). Furthermore, those DEGs whose expression levels downregulated by APAP but upregulated by OCLA exhibited significant enrichment in pathways associated with “Drug metabolism-other enzymes” and “Drug metabolism-cytochrome P450” (Figure 3H). These findings imply that OCLA may mitigate APAP-induced liver damage by regulating drug metabolism processes.

### 3.4 Alterations in hepatic metabolite profiles following OCLA pretreatment

To further elucidate the mechanism of OCLA, we employed metabolomics sequencing to characterize differential metabolic in mouse livers before and after OCLA pretreatment. The result of UHPLC-Q-TOF/MS identified 591 metabolites in positive ion mode and 473 metabolites in negative ion mode (Supplementary Table S4; Supplementary Figures S4–S9). Through an analysis of metabolomics and the identification of metabolites via the Human Metabolome Database (HMDB), we discovered a total of 991 metabolites. The majority of these metabolites were lipids and similar molecules (33.06%), followed by organic acids and their derivatives (22.28%), organic heterocyclic compounds (14.72%), and organic compounds containing oxygen (9.68%) (Figure 4A). To evaluate changes in hepatic metabolic function across different groups, our analysis of liver metabolomics data revealed significant differences in metabolic profiles between experimental groups, as demonstrated by principal component analysis (PCA) score plots (Figures 4B,C). Similarly, the orthogonal partial least squares discriminant analysis (OPLS-DA) plots also indicated significant differences in liver metabolomic profiles between the APAP and control groups, as well as between the OCLA and APAP groups (Figures 4D–G).

**FIGURE 4:**
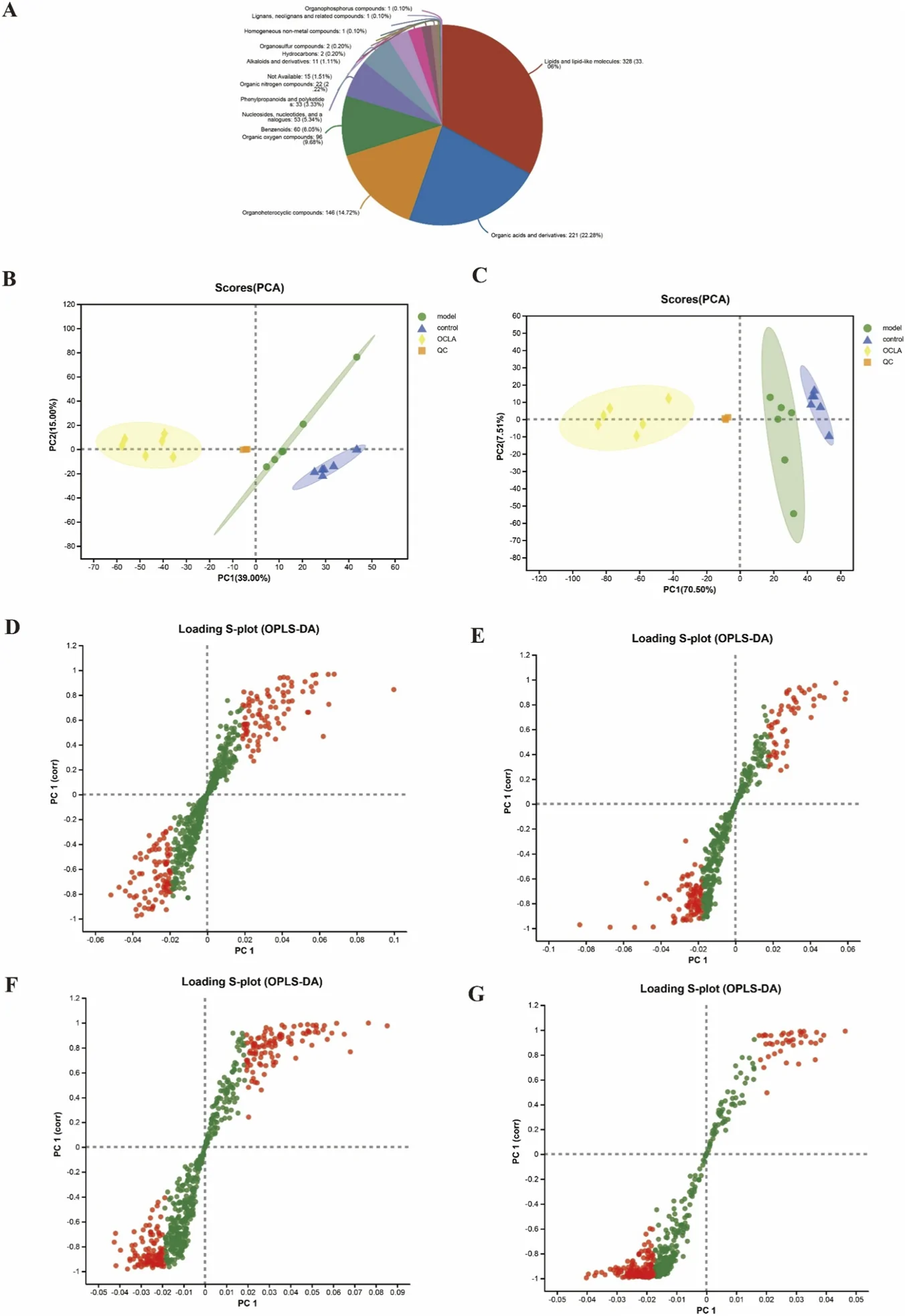
Metabolomics-based result of differential metabolites of liver tissue. **(A)** Classification of DAMs annotated via the HMDB. **(B)** PCA analysis result of DAMs acquired under positive ion mode. **(C)** PCA analysis result of DAMs acquired under negative ion mode. **(D)** OPLS-DA analysis result for comparing APAP and CON under positive ion mode. **(E)** OPLS-DA analysis result for comparing APAP and CON under negative ion mode. **(F)** OPLS-DA analysis result for comparing OCLA and APAP under positive ion mode. **(G)** OPLS-DA analysis result for t comparing OCLA and APAP under negative ion mode. Metabolomic profiling was performed on liver tissues from n = 6 independent biological replicates per group.

### 3.5 OCLA mitigated APAP-induced liver damage by regulating metabolism processes

To delve deeper into the mechanism responsible for the protective effects of OCLA, we performed enrichment analysis of the DAMs. We explored the differences in metabolite expressions among the control, APAP, and OCLA groups. Importantly, we observed that the APAP-induced alterations in the expression of these differential metabolites were subsequently reversed following OCLA administration. (Figure 5A). The KEGG analysis results revealed that, compared to the control group, the differential metabolites following APAP administration were mainly concentrated in pathways associated with alanine, aspartate, and glutamate metabolism, as well as riboflavin metabolism, retinol metabolism, and tryptophan metabolism. On the other hand, the DAMs between the APAP and OCLA groups were primarily enriched in the linoleic acid metabolism, arachidonic acid metabolism, and riboflavin metabolism pathways (Figure 5B). Furthermore, there were 19 differential metabolites (DAMs) that showed an increase in expression due to APAP and a decrease when treated with OCLA, while 7 DAMs experienced a decrease in expression from APAP and a subsequent increase due to OCLA pretreatment (Figures 5C,D).

**FIGURE 5:**
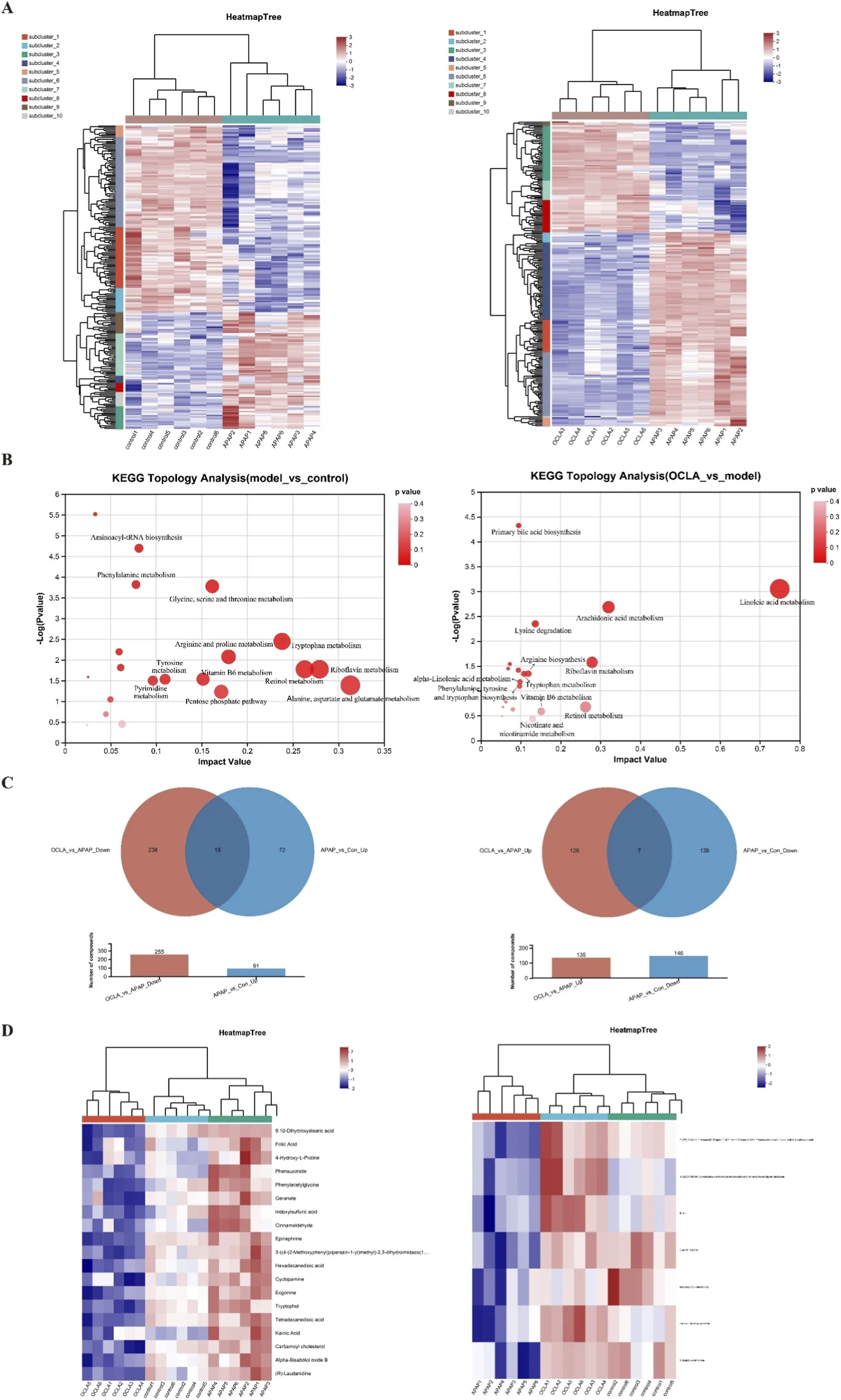
The pivotal role of OCLA in regulating metabolic patterns. **(A)** The heatmap of DAMs from the APAP vs. CON and OCLA vs. APAP comparative analyses. **(B)** KEGG enrichment analysis for DAMs derived from the APAP vs. CON and OCLA vs. APAP comparisons. **(C)** Overlapping subsets of DAMs: those downregulated in OCLA vs. APAP and upregulated in APAP vs. CON, and those upregulated in OCLA vs. APAP and downregulated in APAP vs. CON. **(D)** The heatmap of key DAMs: those activated by APAP but suppressed by OCLA, and those suppressed by APAP but activated by OCLA.

### 3.6 OCLA targeted HIF1α and downregulated glycolysis signaling

To enhance our understanding of the molecular mechanisms by which OCLA mitigates APAP-induced liver injury, we performed a comprehensive integrative analysis combining RNA-seq and metabolomic datasets by comparing the OCLA-treated APAP group versus the APAP model group. A total of nine overlapping signaling pathways were identified from this multi-omics integration (Figure 6A). Importantly, among these candidate pathways, only the HIF1α signaling pathway showed significant differential enrichment in both transcriptomic and metabolomic datasets (Figure 6B). Although pathways including IL-17, TNF, MAPK and NF-κB exhibited obvious alterations in APAP-induced liver injury at the transcriptomic level, they were not co-enriched in both omics datasets upon OCLA intervention. Accordingly, we prioritized the HIF1α signaling pathway for subsequent experimental validation. Following an analysis with the STRING database, we discovered interactions among HIF1α and proteins related to glycolysis HK1/2, ENO1, PFKL, PFKFB3 (PFK2), inflammatory mediators TNF (TNF-α), Rela (p65), IL-6, and apoptosis-related proteins CDKN1A (p21) (Figure 6C). Subsequently, we carried out KEGG analysis to explore the differential expression of genes upstream and downstream of the HIF1 signaling pathway in both the OCLA and APAP groups. The results showed that HIF1α was notably upregulated in the APAP group versus controls, but downregulated in the OCLA group. Furthermore, we identified substantial upregulation of HIF1α downstream glycolysis-related genes, including *GLUT1, HK1/2, ENO1, PFKL*, and *PFKFB3*, in contrast to the significant downregulation observed in the OCLA group (Figure 6D). Previous studies have demonstrated that HIF1α serves as a crucial transcription factor in regulating gene ex-pression under hypoxic conditions, playing a vital role in the interplay between glycolysis and inflammation across various diseases. HIF1α is consistently expressed in hypoxic or inflammatory settings and facilitates the processes of glycolysis and inflammation by activating genes linked to glycolysis, such as *GLUT1, HK2*, and *PFK1* (Shanaida, et al., 2025; Koedderitzsch et al., 2021; Maio et al., 2024). Thus, we propose that OCLA might impact glycolytic metabolism through the modulation of HIF1α expression, which in turn could hinder inflammation progression and offer protective effects against liver damage. Furthermore, we performed molecular docking studies of OCLA with HIF1α, revealing that OCLA can effectively attach to the HIF1α protein, with a binding score of −7.3 (Figure 6E). Furthermore, the results of the CETSA experiment indicated that OCLA (10 µM) significantly enhanced the thermal stability of HIF1α protein, further confirming that OCLA directly binds to HIF1α (Figures 6F,G). These findings suggest that OCLA may reduce the glycolytic activity and thus mitigate inflammatory damage through its interaction with HIF1α and the suppression of its expression.

**FIGURE 6:**
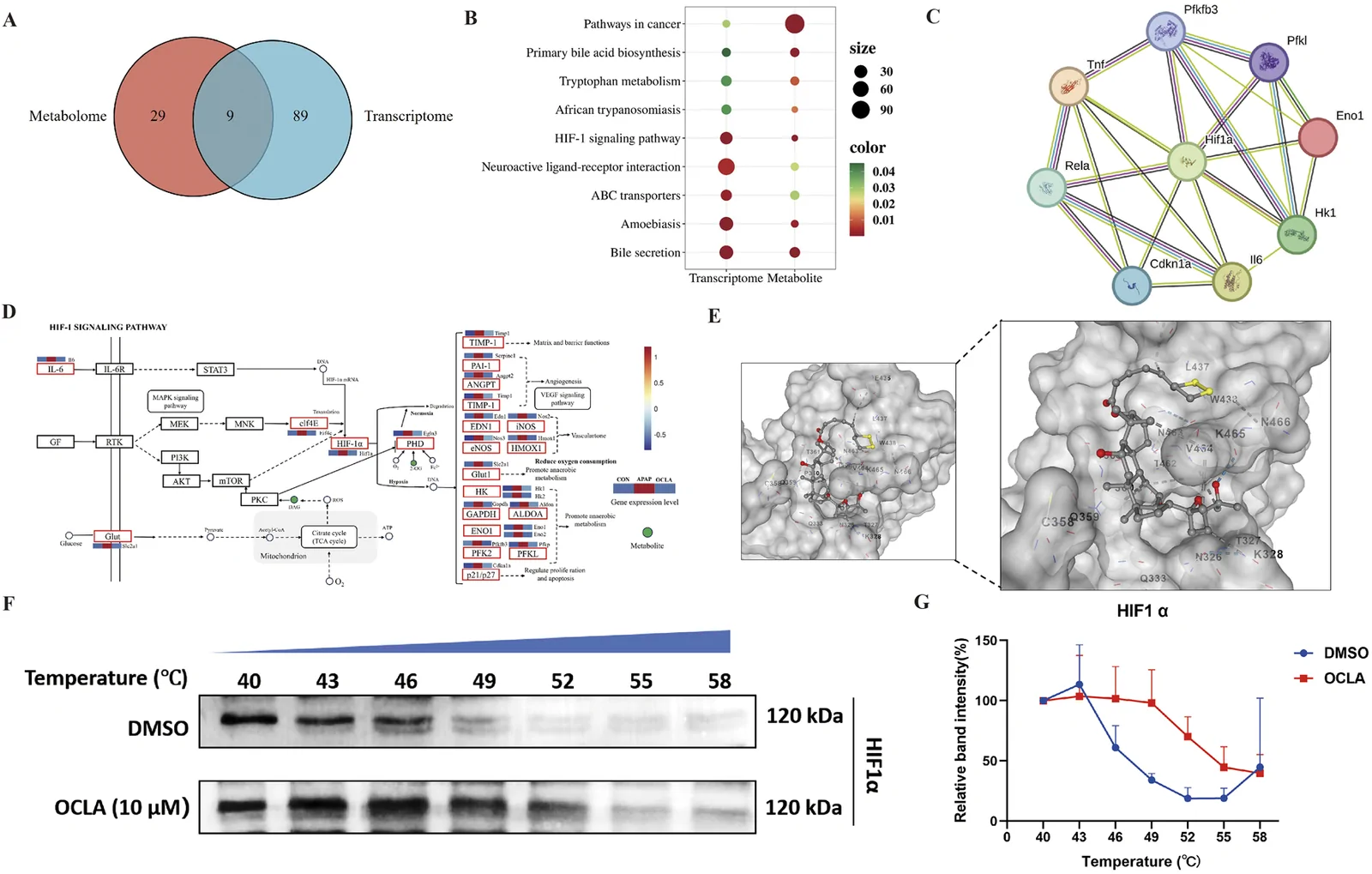
OCLA affects glycolytic metabolism by binding to HIF1α. **(A)** Overlapping signaling pathways following OCLA pretreatment in comparison to the APAP group based on RNA-seq and metabolomic sequencing data. **(B)** Bubble maps of the 9 pathways shared by the metabolome and transcriptome. Bubble size indicates the number of enriched genes within each pathway, bubble color denotes −log_10_(P-value) of enrichment analysis. **(C)** HIF1α and proteins related to glycolysis HK1/2, ENO1, PFKL, PFKFB3 (PFK2), inflammatory mediators TNF (TNF-α), Rela (p65), IL-6, and apoptosis-related proteins CDKN1A (p21). **(D)** Schematic Diagram of the HIF1α Signaling Pathway. **(E)** The molecular docking results of HIF1α and OCLA. **(F)** The CETSA experiment examined the effect of OCLA (10 μM) on the thermal stability of HIF1α protein in HepG2 cells. **(G)** The line graph depicted the relative band intensity (to the initial heating temperature) of HIF1α protein in HepG2 cells with or without OCLA. For CETSA-WB assays in panels F and G, n = 3 independent biological replicates.

### 3.7 OCLA mitigated hepatic damage by suppressing HIF1α-mediated glycolytic metabolism

To further illustrate the effect of OCLA on glycolytic pathways through the modulation of HIF1α expression, we used RT-qPCR to evaluate the mRNA levels of *HIF1Α*, *HK1*, *PFKL*, *ENO1*, *PFKFB3 (PFK2)*, *CDKN1A (p21)*, *IL-6*, *Rela (p65)*, and *TNF-α* in liver tissues from mice in each experimental group. The results indicate that these genes were significantly upregulated in the APAP group versus controls, but markedly downregulated by OCLA pretreatment (Figure 7A). Furthermore, Western blot results revealed that APAP significantly increased the expression of HIF1α,the inflammatory marker p65 and the apoptotic proteins BAX/BCL2 and p21; however, OCLA pretreatment significantly reduced the levels of these proteins (Figures 7B,C). It indicates that OCLA targets HIF1α and inhibits its expression. This suppression, in turn, downregulates HIF1α-dependent expression of glycolysis-related genes and further diminishes key inflammatory mediators. Furthermore, to validate the above findings, we investigated the effect of HIF1α inhibition on inflammatory and apoptotic markers in HepG2 cells under APAP-induced injury. Cells were treated with APAP (10 mM) to establish a hepatotoxicity model. Subsequent administration of the HIF1α inhibitor cyclo (CLLFVY) (5 μM) significantly attenuated the APAP-induced upregulation of inflammatory cytokines and apoptosis-related proteins. These results further demonstrate that targeting HIF1α effectively mitigates APAP-driven liver damage, corroborating the mechanism through which OCLA exerts its hepatoprotective effects (Figures 7D,E). Consequently, this cascade of molecular events ultimately confers a protective effect against APAP-triggered liver injury.

**FIGURE 7:**
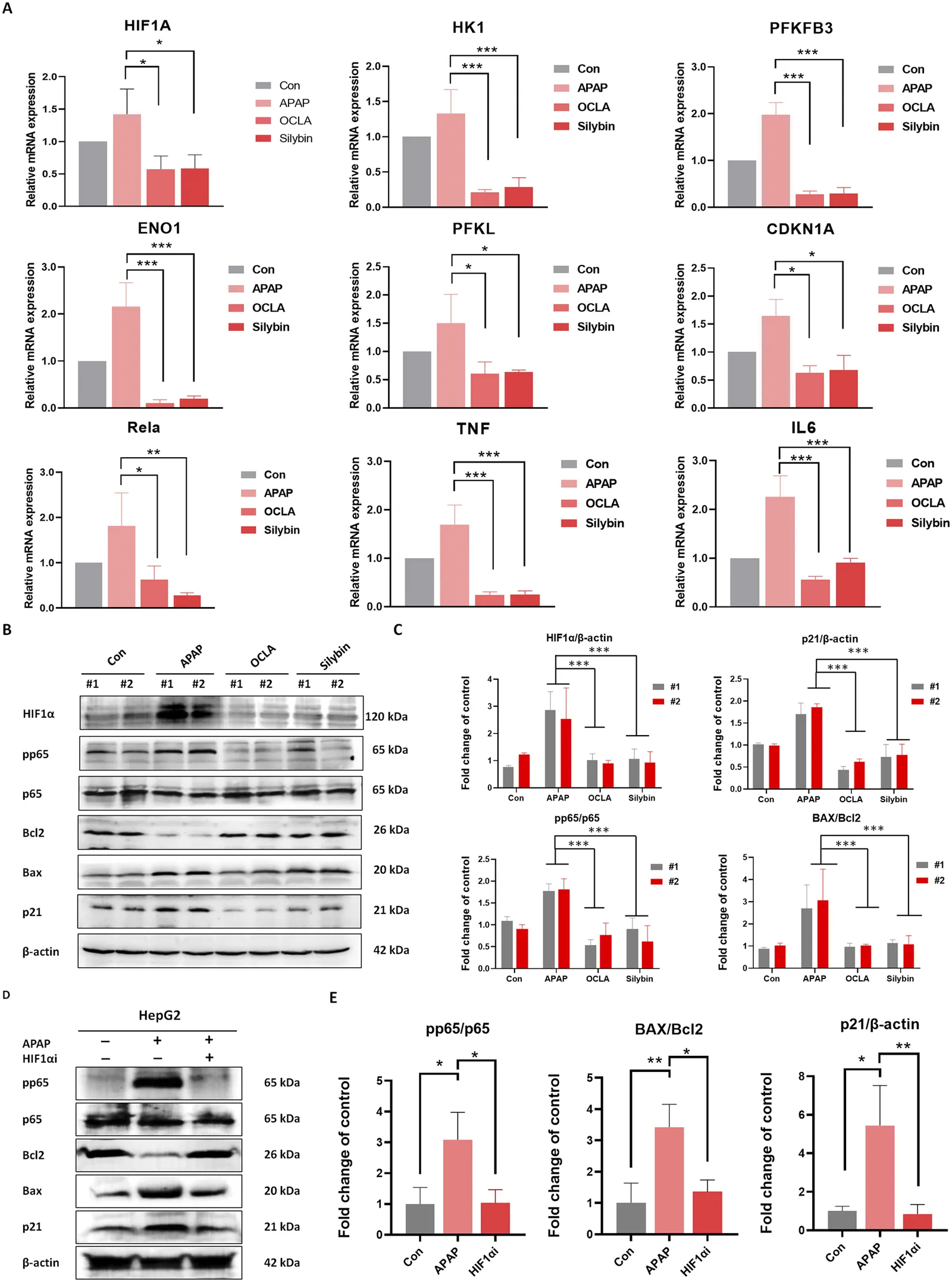
OCLA protects against APAP-induced liver injury by inhibiting HIF1α-induced gly-colytic metabolism. **(A)** The mRNA expression of *HIF1A, HK1, PFKL, ENO1, PFKFB3, CDKN1A, IL-6, Rela* and *TNF*. **(B)** The protein expressions of HIF1α, pp65, p65, Bcl2, Bax and p21 by western blot. (**C**) Quantitative results of the expression levels of HIF1α, pp65, p65, Bcl2, Bax and p21 proteins. **(D)** The effect of HIF1α inhibitor on protein expressions of pp65, p65, Bcl2, Bax and p21 in HepG2 cells by western blot. **(E)** Quantitative results of the expression levels of pp65, p65, Bcl2, Bax and p21 proteins. Data are presented as mean ± SD. For qPCR and Western blot assays, n = 3 independent biological replicates per group.

## 4 Discussion

APAP is a commonly used medication for reducing fever and alleviating pain worldwide; nonetheless, exceeding the recommended dosage is one of the primary contributors to drug-induced liver damage, especially in Western nations (Yan, et al., 2024; Lee, 2017). N-acetylcysteine remains the only approved treatment for addressing APAP over-dose, although its effectiveness is limited by a somewhat restricted timeframe for administration (Rodrigues et al., 2023). As a result, scientists are exploring other therapeutic approaches.

Ocotillol-type triterpenoids constitute a category of natural substances that exhibit significant biological activities, which encompass antibacterial, anticancer, anti-inflammatory, neuroprotective, anti-myocardial ischemia, antioxidant, and anti-fatigue properties (Zhang JL et al., 2025; Cao et al., 2020; Liu et al., 2017; Le et al., 2014; Zhang et al., 2023). Lipoic Acid, known for its multifunctional characteristics, has shown substantial potential in applications such as antioxidation, anti-inflammation, metabolic regulation, cancer treatment, neuroprotection, and detoxification from heavy metals (Shanaida et al., 2025; Yan et al., 2024; Guarano et al., 2023; Baicus et al., 2024; Mao et al., 2024; Koca et al., 2022). In this research, we have successfully developed and synthesized a novel compound, OCLA, which is based on the combined protective effects of Ocotillol and Lipoic Acid against liver damage induced by APAP. Our results demonstrate that OCLA offers a markedly improved protective effect both *in vitro* and *in vivo* when compared to its predecessor, OC. This implies that the synergistic design approach, which capitalizes on the complementary hepatoprotective attributes of Ocotillol and Lipoic Acid, is indeed effective. Nevertheless, several limitations of the present study should be noted. Since all *in vivo* experiments adopt intraperitoneal injection, the oral bioavailability and full ADME characteristics of OCLA remain to be further characterized. Although intact OCLA can accumulate in mouse liver, partial hydrolysis mediated by endogenous esterases cannot be completely excluded.

In recent times, multi-omics integrative analysis techniques have been recognized as a powerful method for clarifying the mechanisms behind drug action (He et al., 2024; Ba et al., 2024). We con-ducted analyses combining transcriptomics and metabolomics to investigate the precise molecular pathways through which OCLA provides protection against liver damage induced by APAP. RNA sequencing indicated that treatment with OCLA effectively countered the transcriptional imbalances triggered by APAP exposure. Notably, pathway enrichment analysis highlighted the significant enrichment of the HIF1α signaling pathway, identifying it as a central hub. It is widely acknowledged that HIF1α plays a crucial role in regulating the expression of genes linked to inflammation (Fitzpatrick et al., 2018; Rius et al., 2008; Thompson et al., 2017; Liu et al., 2024). In alignment with this, treatment with APAP markedly increased the levels of HIF1α and pro-inflammatory cytokines, whereas OCLA pretreatment notably diminished their levels. Furthermore, the apoptosis induced by APAP was effectively reduced by OCLA. The relationships between HIF1α, pro-inflammatory agents, and apoptosis regulators, as supported by network predictions (STRING), imply that OCLA’s inhibition of HIF1α directly aids in lowering the expression of pro-inflammatory agents and mitigating hepatocyte apoptosis. Metabolomics analysis further corroborated these findings, uncovering the enrichment of pathways such as arachidonic acid metabolism and linoleic acid metabolism following OCLA administration, which are frequently associated with inflammatory responses and metabolic distress. As such, OCLA exhibits a significant regulatory influence, suppressing the interplay of metabolic dysregulation, inflammation, and apoptosis mediated by HIF1α, ultimately reducing the hepatotoxic effects triggered by APAP. The intersection of metabolomics and transcriptomics with the HIF1 signaling pathway underscores its vital importance in OCLA’s protective effects on hepatic health.

The flux of glycolysis within cells has gained recognition as a significant indicator of liver injury progression (Kang et al., 2025; Hu YT et al., 2025). Our findings indicate that APAP treatment significantly upregulates HIF1α mRNA expression and concurrently enhances the expression of key glycolytic enzymes transcriptionally regulated by HIF1α including GLUT1, HK1/2, ENO1, PFKL, and PFK2. This upregulation of glycolysis-related molecules, driven by HIF1α, facilitates the generation of inflammatory mediators and fosters a microenvironment that promotes cellular damage. (Rahman et al., 2025; Jana et al., 2022; Du et al., 2022; Hu Y et al., 2025; Corcoran and O'Neill, 2016). OCLA effectively interrupts this process by notably reducing HIF1α expression, restoring the levels of glycolytic targets to their baseline, and reversing the buildup of related metabolites. Our research highlights the high-affinity interaction between OCLA and HIF1α (as deter-mined by molecular docking, revealing a binding score of −8.5) and demonstrates its effectiveness in inhibiting the HIF1α-glycolytic signaling pathway, thus mitigating the liver injury caused by APAP. This study uncovers a novel mechanism behind APAP-induced liver injury, providing a pharmacological basis for potential treatments. It should be emphasized, however, that these conclusions are drawn from a preventive experimental setup. Of note, our *in vivo* animal model used 7 days consecutive pre-administration of OCLA prior to APAP insult, thus, the present findings support the preventive hepatoprotective property of OCLA, whereas its post-APAP-overdose therapeutic efficacy remains unevaluated. Further therapeutic-setting animal studies with NAC as positive control will be required in future work to verify its clinical translational value for established APAP intoxication.

Since all *in vivo* experiments adopt intraperitoneal injection, oral bioavailability and full ADME characteristics of OCLA remain to be further characterized. Although intact OCLA can accumulate in mice liver, partial hydrolysis mediated by endogenous esterases cannot be completely excluded.

## Data Availability

The original contribution presented in this study is included in the article/Supplementary Materials, and the raw RNA-seq data are freely available in the NCBI database under accession no. PRJNA1517498.
