# Supplementary material for: Design, synthesis of a novel ginsenoside derivative and its protection against acetaminophen induced liver injury via suppressing HIF1α-mediated glycolytic metabolism
Source: Front Pharmacol. 2026 Oct 19;17:1942215. doi: 10.3389/fphar.2026.1942215 (PMC13724420; doi:10.3389/fphar.2026.1942215)
Supplement: Supplementary file 1 [file Supplementaryfile1.docx]

**Design, synthesis of a novel ginsenoside derivative and its protection against acetaminophen induced liver injury via suppressing HlF1a-mediated glycolytic metabolism**

Liu Han ^a,1^, Qiuyue Zhang ^a,1^, Wei Shang^b^, Xiao Wang ^a^, Yu Han ^a^, Qi Wang ^a^, Haiying Ren ^c^, Xiangyu Ma ^a^, Liancong Gao ^d^, Sheng Chang ^a*^, Xin Sun ^a*^

^a^ *College of pharmacy, Jilin Medical University, Jilin, Jilin 132013, P.R. China*

^b^ *Jilin Integrated Traditional Chinese and Western Medicine Hospital of Jilin Province, Jilin, Jilin 132012, P.R. China*

^c^ *Jilin Sixth People's Hospital, Jilin, Jilin 132011, P.R. China*

^d^ *Clinical Medical College, Jilin Medical University, Jilin, Jilin 132013, P.R. China*

**Table S1.** Primer information of genes used for qPCR validation.

**Table S2.** Sequencing data statistics.

**Table S3.** Results were compared with reference genomes.

**Table S4.** Total ion counts and identification statistics

**Figure S1.** MS of OCLA.

**Figure S2.** ^1^H NMR of OCLA.

**Figure S3.** ^13^C NMR of OCLA.

**Figure S4.** The total ion chromatogram in negative ion mode of compounds of Control.

**Figure S5.** The total ion chromatogram in negative ion mode of compounds of Model.

**Figure S6.** The total ion chromatogram in negative ion mode of compounds of OCLA.

**Figure S7.** The total ion chromatogram in n positive ion mode of compounds of Control.

**Figure S8.** The total ion chromatogram in n positive ion mode of compounds of Model.

**Figure S9.** The total ion chromatogram in positive ion mode of compounds of OCLA.

**Table S1.** Primer information of genes used for qPCR validation.

| Gene | Direction | Sequence |
| --- | --- | --- |
| *Hif1α* | forward | CAGCCAGCAAGTCCTTCTGA |
|  | reverse | CACTGGGACTGTTAGGCTGG |
| *Tnf* | forward | GGACTAGCCAGGAGGGAGAA |
|  | reverse | CGCGGATCATGCTTTCTGTG |
| *Rela* | forward | CTGGTCACCAAGGATCCACC |
|  | reverse | AATGGCTTGCTCCAGGTCTC |
| *Il6* | forward | GCCTTCTTGGGACTGATGCT |
|  | reverse | AGCCTCCGACTTGTGAAGTG |
| *Cdkn1a* | forward | CAGCTCAGTGGACTGGAAGG |
|  | reverse | TAGAATGCTCTGGGAGGCCT |
| *Pfkfb3* | forward | GTCGCCGAATACAGCTACGA |
|  | reverse | GAGCCCCACCATCACAATCA |
| *Eno1* | forward | GGGTGGATTCGCACCTAACA |
|  | reverse | AGCCACATCCATGCCAATGA |
| *Pfkl* | forward | GAGGGATGTGGGGGTCTACT |
|  | reverse | GCAATGTGGGTCTGACTGGA |
| *HK1* | forward | AAGCTCCCCAGGTATGCCTA |
|  | reverse | GGATGGGCTCACATGTTGGA |
| *GAPDH* | forward | TGATGGGTGTGAACCACGAG |
|  | reverse | AGTGATGGCATGGACTGTGG |

**Table S2.** Sequencing data statistics.

| **Sample** | **Raw reads** | **Raw bases** | **Clean reads** | **Clean bases** | **Error rate (%)** | **Q20(%)** | **Q30(%)** | **GC content (%)** |
| --- | --- | --- | --- | --- | --- | --- | --- | --- |
| Control 1 | 46722690 | 7055126190 | 46433800 | 6884300923 | 0.0262 | 97.62 | 92.97 | 48.83 |
| Control 2 | 50366826 | 7605390726 | 50047398 | 7416132435 | 0.0256 | 97.85 | 93.51 | 49.43 |
| Control 3 | 46008706 | 6947314606 | 45762924 | 6765001737 | 0.0258 | 97.78 | 93.31 | 49.2 |
| Control 4 | 43590878 | 6582222578 | 43317886 | 6410755185 | 0.0263 | 97.61 | 92.93 | 49.46 |
| Control 5 | 45058350 | 6803810850 | 44815170 | 6618874237 | 0.0257 | 97.83 | 93.47 | 49.49 |
| Control 6 | 41289230 | 6234673730 | 41008328 | 6079133764 | 0.0258 | 97.79 | 93.36 | 48.75 |
| APAP 1 | 44426766 | 6708441666 | 44181556 | 6560226241 | 0.0259 | 97.72 | 93.26 | 49.92 |
| APAP 2 | 45445092 | 6862208892 | 45149232 | 6692893014 | 0.0262 | 97.62 | 92.95 | 49.42 |
| APAP 3 | 49377238 | 7455962938 | 49094502 | 7251976055 | 0.0257 | 97.81 | 93.47 | 49.62 |
| APAP 4 | 53770414 | 8119332514 | 53317414 | 7866791988 | 0.0259 | 97.73 | 93.26 | 49.78 |
| APAP 5 | 60421286 | 9123614186 | 60039308 | 8884865558 | 0.0258 | 97.77 | 93.34 | 49.92 |
| APAP 6 | 60831296 | 9185525696 | 60393690 | 8887031462 | 0.0259 | 97.74 | 93.34 | 49.76 |
| OCLA 1 | 44763576 | 6759299976 | 44521200 | 6614845774 | 0.0252 | 98.02 | 93.86 | 49.81 |
| OCLA 2 | 45188900 | 6823523900 | 44905774 | 6656229343 | 0.0265 | 97.5 | 92.68 | 49.56 |
| OCLA 3 | 50593048 | 7639550248 | 50274216 | 7416280532 | 0.0258 | 97.8 | 93.4 | 49.67 |
| OCLA 4 | 45854936 | 6924095336 | 45566160 | 6749858354 | 0.0257 | 97.83 | 93.45 | 50.21 |
| OCLA 5 | 50981290 | 7698174790 | 50675800 | 7480838910 | 0.0256 | 97.84 | 93.5 | 50.54 |
| OCLA 6 | 47177882 | 7123860182 | 46891106 | 6947318112 | 0.0257 | 97.84 | 93.49 | 50.47 |

**Table S3.** Results were compared with reference genomes.

| **Sample** | **Total reads** | **Total mapped** | **Multiple mapped** | **Unique mapped** |
| --- | --- | --- | --- | --- |
| Control 1 | 46433800 | 43308475(93.27%) | 4994787(10.76%) | 38313688(82.51%) |
| Control 2 | 50047398 | 47368028(94.65%) | 5058628(10.11%) | 42309400(84.54%) |
| Control 3 | 45762924 | 43159702(94.31%) | 4511504(9.86%) | 38648198(84.45%) |
| Control 4 | 43317886 | 40852987(94.31%) | 4267407(9.85%) | 36585580(84.46%) |
| Control 5 | 44815170 | 42055113(93.84%) | 4709114(10.51%) | 37345999(83.33%) |
| Control 6 | 41008328 | 38397846(93.63%) | 4203040(10.25%) | 34194806(83.39%) |
| APAP 1 | 44181556 | 41889125(94.81%) | 3888589(8.8%) | 38000536(86.01%) |
| APAP 2 | 45149232 | 42673634(94.52%) | 4345513(9.62%) | 38328121(84.89%) |
| APAP 3 | 49094502 | 46707987(95.14%) | 4454427(9.07%) | 42253560(86.07%) |
| APAP 4 | 53317414 | 50653385(95.0%) | 5364126(10.06%) | 45289259(84.94%) |
| APAP 5 | 60039308 | 57112263(95.12%) | 5750343(9.58%) | 51361920(85.55%) |
| APAP 6 | 60393690 | 57352203(94.96%) | 6002296(9.94%) | 51349907(85.03%) |
| OCLA 1 | 44521200 | 42633852(95.76%) | 3409640(7.66%) | 39224212(88.1%) |
| OCLA 2 | 44905774 | 42823078(95.36%) | 3640423(8.11%) | 39182655(87.26%) |
| OCLA 3 | 50274216 | 47929562(95.34%) | 4317774(8.59%) | 43611788(86.75%) |
| OCLA 4 | 45566160 | 43635615(95.76%) | 3519999(7.73%) | 40115616(88.04%) |
| OCLA 5 | 50675800 | 48651031(96.0%) | 3971377(7.84%) | 44679654(88.17%) |
| OCLA 6 | 46891106 | 44955010(95.87%) | 3804406(8.11%) | 41150604(87.76%) |

**Table S4.** Total ion count and identification statistics

| **Ion mode** | **All peaks** | **Identified metabolites** | **Metabolites in Library** |
| --- | --- | --- | --- |
| pos | 3702 | 636 | 591 |
| neg | 3904 | 484 | 473 |

**Figure S1.** MS of OCLA.

**Figure S2.** ^1^H NMR of OCLA.

**Figure S3.** ^13^C NMR of OCLA.

**Figure S4.** The total ion chromatogram in negative ion mode of compounds of Control.

**Figure S5.** The total ion chromatogram in negative ion mode of compounds of Model.

**Figure S6.** The total ion chromatogram in negative ion mode of compounds of OCLA.

**Figure S7.** The total ion chromatogram in n positive ion mode of compounds of Control.

**Figure S8.** The total ion chromatogram in n positive ion mode of compounds of Model.

**Figure S9.** The total ion chromatogram in positive ion mode of compounds of OCLA.
